# ALKBH5 suppresses tumor progression via an m^6^A-dependent epigenetic silencing of pre-miR-181b-1/YAP signaling axis in osteosarcoma

**DOI:** 10.1038/s41419-020-03315-x

**Published:** 2021-01-11

**Authors:** Ye Yuan, Gege Yan, Mingyu He, Hong Lei, Linqiang Li, Yang Wang, Xiaoqi He, Guanghui Li, Quan Wang, Yuelin Gao, Zhezhe Qu, Zhongting Mei, Zhihua Shen, Jiaying Pu, Ao Wang, Wei Zhao, Huiwei Jiang, Weijie Du, Lei Yang

**Affiliations:** 1 Department of Orthopedics at The First Affiliated Hospital, and Department of Pharmacology at College of Pharmacy (The Key Laboratory of Cardiovascular Medicine Research, Ministry of Education), Harbin Medical University, 150086 Harbin, China; 2 Department of Pharmacy, The Second Affiliated Hospital of Harbin Medical University, 150086 Harbin, China; 3 Department of Clinical pharmacology, College of Pharmacy, Harbin Medical University, 150086 Harbin, China; 4 Research Unit of Noninfectious Chronic Diseases in Frigid Zone, Chinese Academy of Medical Sciences, 2019RU070 Harbin, China; 5 Department of General Surgery, The First Affiliated Hospital of Harbin Medical University, 150001 Harbin, China

**Keywords:** Sarcoma, Apoptosis, Gene silencing

## Abstract

ALKBH5 is the main enzyme for m^6^A-based demethylation of RNAs and it has been implicated in many biological and pathophysiological processes. Here, we aimed to explore the potential involvement of ALKBH5 in osteosarcoma and decipher the underlying cellular/molecular mechanisms. We discovered downregulated levels of demethylase ALKBH5 were correlated with increased m^6^A methylation in osteosarcoma cells/tissues compared with normal osteoblasts cells/tissues. ALKBH5 overexpression significantly suppressed osteosarcoma cell growth, migration, invasion, and trigged cell apoptosis. In contrast, inhibition of ALKBH5 produced the opposite effects. Whereas ALKBH5 silence enhanced m^6^A methylations of pre-miR-181b-1 and YAP-mRNA exerting oncogenic functions in osteosarcoma. Moreover, upregulation of YAP or downregulation of mature miR-181b-5p displayed a remarkable attenuation of anti-tumor activities caused by ALKBH5. Further results revealed that m^6^A methylated pre-miR-181b-1 was subsequently recognized by m^6^A-binding protein YTHDF2 to mediate RNA degradation. However, methylated YAP transcripts were recognized by YTHDF1 to promote its translation. Therefore, ALKBH5-based m^6^A demethylation suppressed osteosarcoma cancer progression through m^6^A-based direct/indirect regulation of YAP. Thus, ALKBH5 overexpression might be considered a new approach of replacement therapy for osteosarcoma treatment.

## Introduction

Osteosarcoma is one of the most common primary solid malignancy of bone, primarily affecting teenagers and young adults^1,2^. Standard treatments for patients include chemotherapy and surgery. Survival has increased considerably due to the advanced treatment strategies^3^, however, there is still no known way to prevent it. Thus, it is urgent to get insight into the underlying mechanism, and develop new therapeutic agents against osteosarcoma.

N^6^-methyladenosine (m^6^A) is an abundant modification of messenger RNAs (mRNAs) in eukaryotes^4–6^. The effects of m^6^A modification on RNA are determined by the interplay between m^6^A methyltransferases (writers), demethylases (erasers), and binding proteins (readers). Recently, the key components of m^6^A writers have been identified including a stable heterodimer core complex of methyltransferase-like 3—methyltransferase-like 14 (METTL3-METTL14) that functions in cellular m^6^A deposition on mammalian nuclear RNAs, as well as Wilms’ tumor 1-associating protein (WTAP), as a splicing factor, interacting with this complex and affecting this methylation^7,8^. While, m^6^A erasers including fat mass and obesity-associated protein (FTO) and ALKB homolog 5 (ALKBH5) remove m^6^A modification from RNA, which interacts with m^6^A readers such as YTH N6-methyladenosine RNA binding protein 1 (YTHDF1) and insulin-like growth factor 2 mRNA binding protein 1 (IGF2BP1)^9^ ect. It has been reported m^6^A based modification exerts diverse biological functions^10–14^. For instance, FTO acts as an oncogenic factor in acute myeloid leukemia (AML)^15^. ALKBH5 has been shown to be involved in pancreatic cancer^16^, glioblastoma^17^, and to impacts male mouse fertility^18^. However, it remains largely unknown the functions and underlying mechanism of m^6^A modification in human osteosarcoma.

Here, we reported that ALKBH5-induced m^6^A demethylation inhibits human osteosarcoma tumor cell growth, migration, and invasion through m^6^A-based post-transcriptional regulation of pre-miRNA-181b-1 and an oncogenic transcriptional co-activator Yes-associated protein 1 (YAP).

## Results

### The m^6^A demethylase ALKBH5 is downregulated in human osteosarcoma

**Fig. 1:**
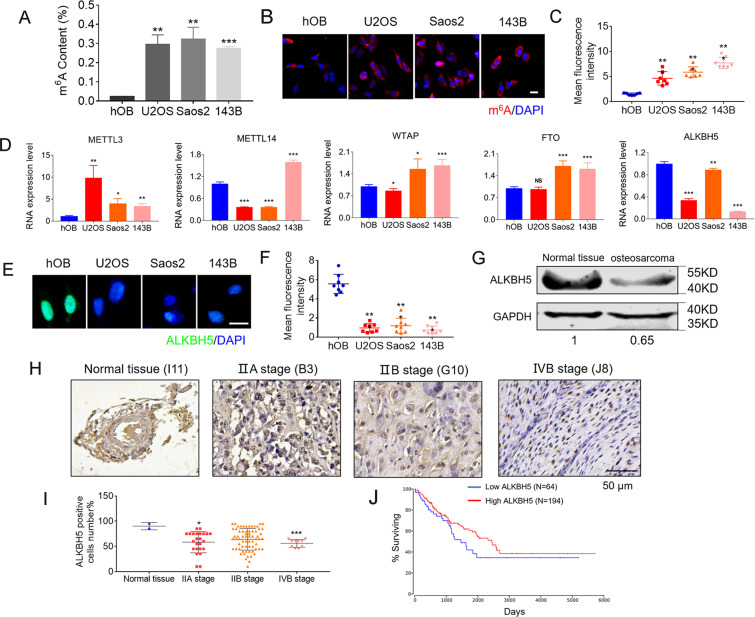
Increased m^6^A modification level together with the reduced expression of demethylase ALKBH5 in human osteosarcoma. **A** m^6^A ELISA experiments (*n* = 3) showing the increase of global m^6^A level in RNAs in human osteosarcoma cell lines compared with human osteoblasts (hOB) cell line. **B** Representative confocal microscopy images with m^6^A (red) and DAPI (blue) of human osteosarcoma cells compared with hOB cells (*n* = 7, Bar: 25 μm). **C** Bar graph showing the quantification of mean influence intensity of m^6^A positive cells. **D** Expression of individual m^6^A modifiers in human osteosarcoma cells compared with hOB cells. **E** Immunostaining for anti-ALKBH5 in hOB cells and osteosarcoma cells (*n* = 8, Bar: 25 μm). **F** Bar graph showing the quantification of mean influence intensity of ALKBH5 positive cells. **G** Western blot showing the protein expression of ALKBH5 in normal tissues and osteosarcoma tissues. **H** Immunohistochemistry (IHC) analysis of ALKBH5 protein expression on tissue microarrays (TMAs) composed of benign bone tissues (*n* = 2), IIA stage osteosarcomas (*n* = 24), IIB stage osteosarcomas (*n* = 66) and IVB stage osteosarcomas (*n* = 10) tumor cores. Representative IHC images (magnification ×80) are presented (upper, Bar: 50 μm). **I** Bar graph representing the percentages of ALKBH5 positive cells number (lower). **J** Kaplan–Meier survival curve indicates the difference in survival rate between ALKBH5 high expression and ALKBH5 low expression patients. Data are expressed as mean ± SEM. *P < 0.05; **P < 0.01; ***P < 0.001.

We firstly quantified m^6^A contents by m^6^A ELISA and immunofluorescence (IF) assays in human osteosarcoma cell lines such as U2OS, Saos2, 143B, and human osteoblast (hOB) hFOB1.19 cell line. The results showed that m^6^A contents were significantly increased in osteosarcoma cells (Fig. 1A–C). Moreover, there was a significant decrease in demethylase ALKBH5 mRNA inversely correlated with m^6^A contents in all three osteosarcoma cell lines as compared with hOB cells, but not in METTL3, METTL14, WTAP, and FTO (Fig. 1D). Meanwhile, immunostaining confirmed a significant decrease of ALKBH5 in U2OS, Saos2, 143B osteosarcoma cell lines, as compared with hOB cells (Fig. 1E, F). Furthermore, lower protein expression of ALKBH5 was detected in human osteosarcoma tissues as compared with normal bone tissues (Fig. 1G). We further applied immunohistochemistry (IHC) assays to measure ALKBH5 protein expression in osteosarcoma tissue microarrays (TMAs) containing 102 tissue cores (Fig. 1H, I and Supplementary Fig. S1). Significantly lower ALKBH5 protein expression was detected in malignant osteosarcoma cores especially in the IVB stage, the highest degree of osteosarcoma, compared with normal bone tissues (Fig. 1H, I). Kaplan–Meier survival analysis from The Cancer Genome Atlas (TCGA) data set (http://www.oncolnc.org/) showed that patients with high ALKBH5 expression exhibited a superior survival, while patients with low ALKBH5 expression exhibited poor survival rate (Fig. 1J). The above results demonstrated that ALKBH5 is generally downregulated and may mediate m^6^A modification having predominant roles in human osteosarcoma.

### ALKBH5-dependent m^6^A demethylation of RNAs severely impacted the growth and motility of osteosarcoma cells

**Fig. 2:**
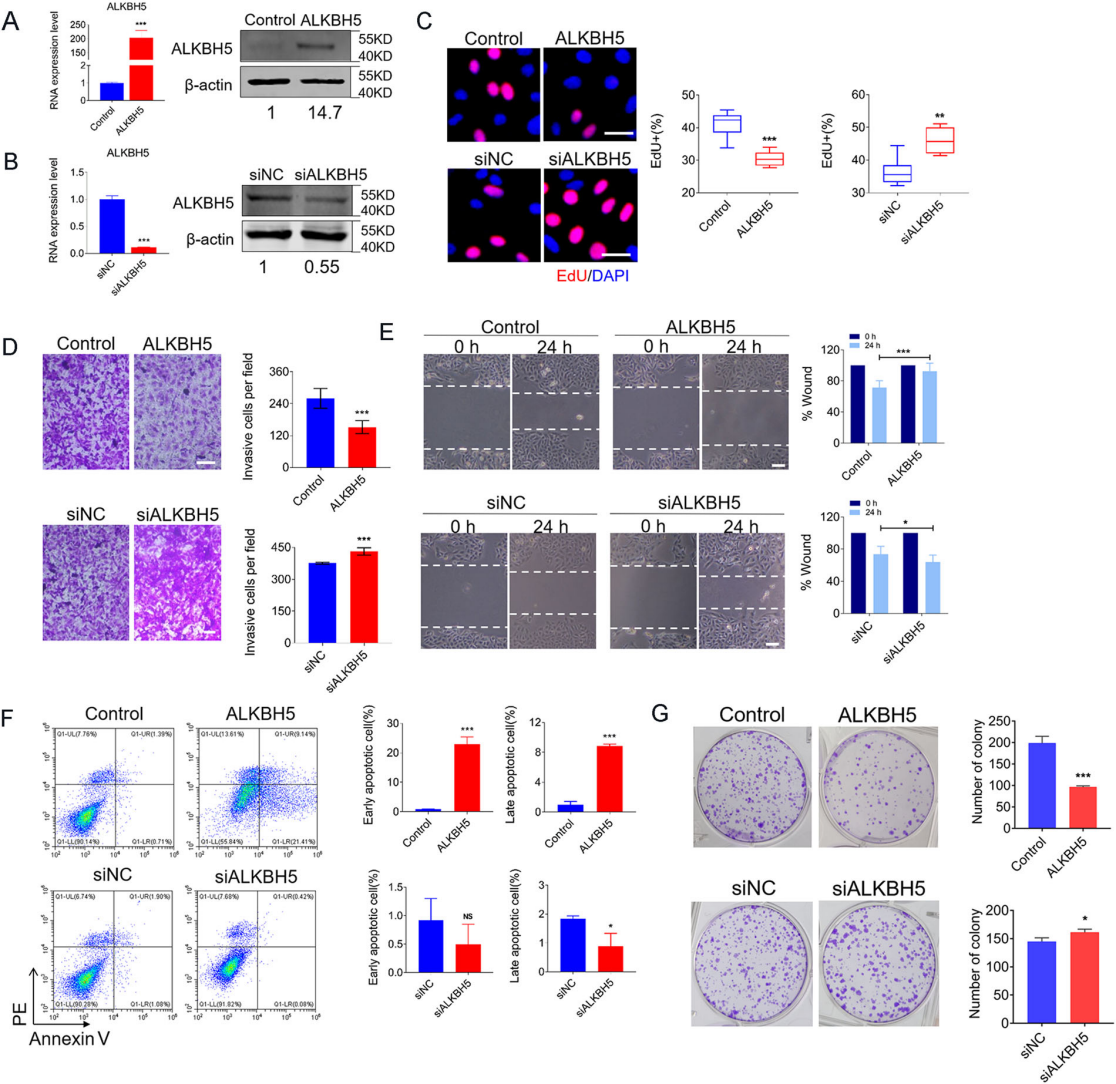
Tumor inhibition impact of ALKBH5 in human osteosarcoma cells. **A**, **B** qRT-PCR (*n* = 3) and western blot were performed to confirm the transfection efficiency of ALKBH5 in U2OS cells. **C** Effects of forced expression of ALKBH5 (upper) and ALKBH5 silencing (lower) on U2OS cell proliferation were tested by EdU staining (Bar: 25 μm, *n* = 3). **D** Transwell assays showing the invasion ability (Bar: 150 μm, *n* = 4). Bar graph representing the quantification of invasive cells. **E** Migration ability was detected by wound-healing assay at 0 and 24 h, respectively, after transfection. Bar graph displaying the mean relative distance of migrated cells (Bar: 200 μm, *n* = 5). **F** Annexin V-FITC/PI staining analysis of cell apoptosis after transfection of ALKBH5 plasmid or siRNA for 24 h. **G** The effects of ALKBH5 on colony-formation ability. (*n* = 3). Bar graph illustrating the quantitative analysis. Data are expressed as mean ± SEM. *P < 0.05; **P < 0.01; ***P < 0.001.

To determine whether ALKBH5 regulated m^6^A modification has a role in osteosarcoma cells, we conducted gain-of-function and loss-of-function studies. As depicted in Fig. 2A, B, the transfection efficiency of ALKBH5 plasmids or siRNA was confirmed by qRT-PCR and western blot. Next, we examined the effect of ALKBH5 on cell proliferation, migration, and invasion. Indeed, overexpressed ALKBH5 remarkably inhibited cell proliferation, invasion, and migration abilities in U2OS cells in both EdU staining, wound-healing cell migration, and the Transwell cell invasion assays, while inhibition of ALKBH5 induced the opposite effects (Fig. 2C–E). In addition, the percentage of both early and late apoptotic cells based on Annexin V/PI staining were significantly increased upon overexpression of ALKBH5, while little effects were observed on ALKBH5 knockdown (Fig. 2F). Moreover, elevated ALKBH5 decreased, or depleting ALKBH5 increased the colony-formation capacities of U2OS osteosarcoma cells (Fig. 2G). In line with the results for U2OS, these effects of ALKBH5 were further confirmed in another osteosarcoma cell line Saos2 (Supplementary Fig. S2).

### Identification of ALKBH5/m^6^A―pre-miR-181b-1/miR-181b-5p―YAP axis as a novel pathway leading to osteosarcoma tumor suppression

**Fig. 3:**
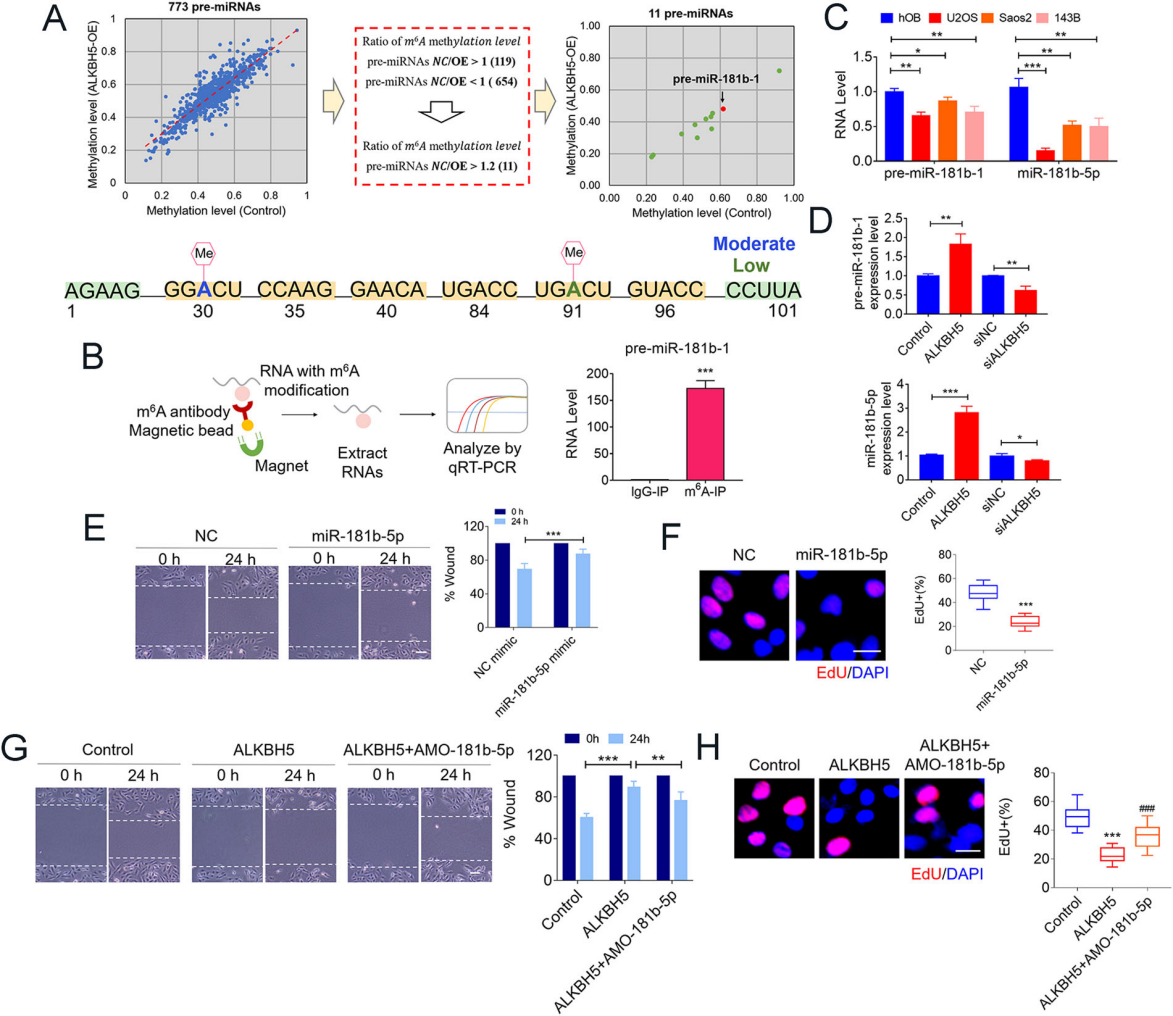
ALKBH5 weakens the m^6^A methylation modification of pre-miR-181b-1 and enhances the expression levels of both pre-miR-181b-1 and miR-181-5p. **A** m^6^A-RIP microarray analysis (upper) showing inhibitory effects of ALKBH5 on m^6^A methylation of pre-miRNAs relative to the control group. Two potentially m^6^A sites of pre-miR-181b-1 predicted by SRAMP program (lower). **B** m^6^A methylation modification of miR-181-5p detected by gene-specific m^6^A assay. **C** pre-miR-181b-1 and miR-181-5p endogenous levels in osteosarcoma cell lines compared with hOB cells. **D** qRT-PCR analysis revealed the function of ALKBH5 overexpression or knockdown on pre-miR-18b-1 and miR-181-5p expression. **E** Wound-healing assay performed at 0 and 24 h, respectively, after transfected with NC or miR-181-5p mimics. Bar graph representing mean relative distance of migrated cells (Bar: 200 μm, *n* = 4). **F** Representative images of EdU staining in U2OS cells with or without miR-181-5p mimics. Bar graph quantifying the percentage of EdU-positive cells (Bar: 25 μm, *n* = 5). **G** Migration ability of U2OS after transfected with ALKBH5 plasmids and/or co-transfected with miR-181-5p inhibitor (AMO-181-5p) (Bar: 200 μm, *n* = 4). **H** EdU staining showing the reversing effects of AMO-181-5p on cell proliferation (Bar: 25 μm, *n* = 5). Data are expressed as mean ± SEM. *P < 0.05; **P < 0.01; ***P < 0.001 (vs. the first group). ^###^*P* < 0.001 (vs. the second group).

As shown above, we have demonstrated the importance of ALKBH5-dependent m^6^A demethylation of RNAs for osteosarcoma tumor suppression with both gain- and loss-of-function approaches. Next, we went on to get further insight into the mechanisms accounting for our findings. It has been reported that in addition to protein-coding genes, large non-coding RNAs function as gene regulators during the progression of bone cancer^19,20^. MiRNA processing is also regulated specifically for osteosarcoma. However, no reports have shown the biological function of m^6^A modification during miRNA processing in osteosarcoma. Therefore, we used m^6^A epitranscriptomic microarray to identify the modified miRNAs precursor of ALKBH5 in control- and overexpressed ALKBH5-transfected U2OS cells (Fig. 3A). Of 773 pre-miRNAs detected by the microarray, we identified 11 pre-miRNAs with >20% decrease (>1.2-fold decrease) in ALKBH5 overexpression-treated cells relative to those in the control cells. The top 10 ALKBH5-mediated m^6^A-demethylated pre-miRNAs are listed in Table 1. Notably, among the pre-miRNAs, pre-miR-181b-1 methylation was markedly decreased upon overexpression of ALKBH5. More importantly, pre-miR-181b-1 sequences are broadly conserved across species. These findings suggest pre-miR-181b-1 as a potential target for ALKBH5 actions in osteosarcoma. Next, we confirmed ALKBH5 overexpression effects on m^6^A level changes of pre-miR-181b-1 using gene-specific m^6^A-qPCR. As shown in Fig. 3B, we observed a strong enrichment of pre-miR-181b-1 in the m^6^A-RIP but not in the IgG-IP fractions. In addition, both pre-miR-181b-1 and mature miR-181b-5p was much lower in osteosarcoma cells than in hOB cells (Fig. 3C). Overexpression of ALKBH5 produced significant increases in the expression levels of both pre-miR-181b-1 and miR-181b-5p in U2OS cells. On the contrary, inhibition of ALKBH5 produced the opposite effects (Fig. 3D). As we expected, miR-181b-5p resulted in a decrease in cell migration (Fig. 3E) and cell proliferation (Fig. 3F). Moreover, downregulation of miR-181b-5p (AMO-181b-5p) partly rescued the decreased cell migration and proliferation caused by ALKBH5 overexpression in U2OS cells (Fig. 3G, H).

**Table 1:** Top 10 pre-miRNAs with ALKBH5-mediated m^6^A demethylation.

Gene symbol	Control (IP)	ALKBH5 (IP)	Control (supernatant)	ALKBH5 (supernatant)	Control (methylation level)	ALKBH5 (methylation level)	Fold change (control/ALKBH5)	Conserved
pre-miR-3182	−2.73	−2.62	−2.60	−1.40	0.48	0.30	1.59	Poorly
pre-miR-890	−4.66	−6.51	−4.97	−5.66	0.55	0.36	1.55	Poorly
pre-miR-1260b	−1.20	−2.05	−1.50	−1.65	0.55	0.43	1.28	Poorly
pre-miR-181b-1	−**4.07**	−**5.45**	**−4.75**	−**5.35**	**0.62**	**0.48**	**1.28**	**Yes**
pre-miR-4779	−0.99	−4.93	−4.54	−6.31	0.92	0.72	1.28	Poorly
pre-miR-27a	−4.63	−0.17	−2.88	2.01	0.23	0.18	1.27	Yes
pre-miR-6763	−4.23	−5.84	−4.36	−5.37	0.52	0.42	1.25	Poorly
pre-miR-1208	−4.73	−5.57	−3.05	−3.50	0.24	0.19	1.24	Poorly
pre-miR-4465	−4.63	−6.25	−4.98	−5.99	0.56	0.45	1.23	Poorly
pre-miR-4293	−5.28	−5.63	−5.07	−4.94	0.46	0.38	1.21	Poorly

*pre-miRNA or pre-miR* precursor miRNA, *IP* immunoprecipitation. Bold values: the target gene having highly conserved.

**Fig. 4:**
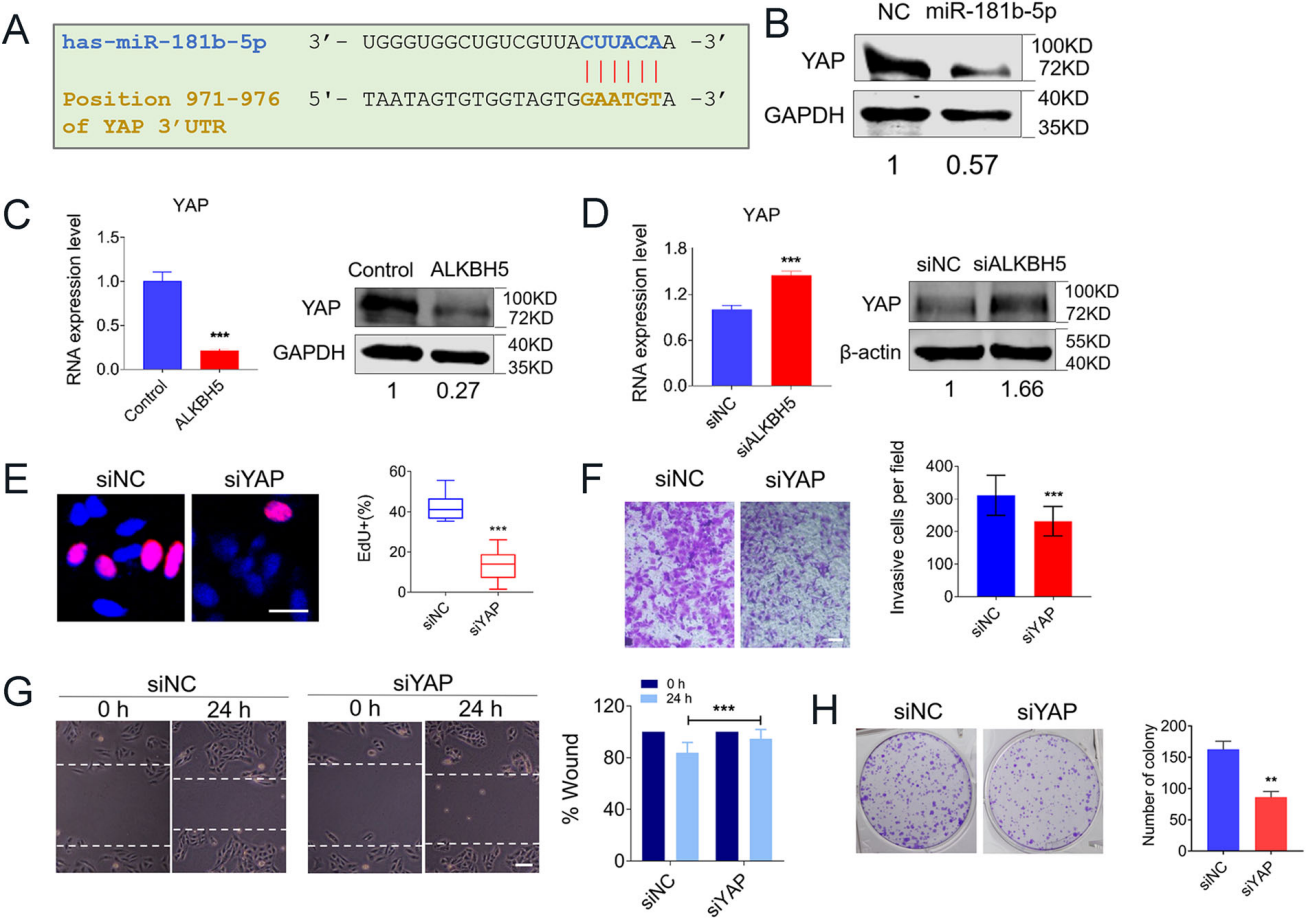
YAP is the critical target gene of miR-181-5p in human OS cells. **A** Sequence alignment showing the complementarity between miR-181-5p and YAP gene with the potential binding sites (seed site). The red bases indicate the seed site and the vertical lines represent the base-pairing between miR-181-5p and YAP. **B** The change of YAP-protein level after transfection of miR-181-5p mimics. **C** qRT-PCR (*n* = 3, left) and western blot (right) showing the decreased expression of YAP with ectopic expression of ALKBH5 (*n* = 3). **D** Expression of YAP at mRNA (left) and protein (right) levels with or without ALKBH5 silence. **E** EdU staining for evaluation of the YAP knockdown influences on the proliferation of U2OS cells (Bar: 25 μm, *n* = 5). **F** Representative images of invasive cells on the membrane for Transwell assay (Bar: 150 μm, *n* = 4). **G** Migration ability was detected by wound-healing assay at 0 and 24 h, respectively, with or without YAP silencing. (Bar: 200 μm, *n* = 5). **H** Representative images of the colony-formation assay with or without YAP silencing. (*n* = 3). Data are expressed as mean ± SEM. **P < 0.01; ***P < 0.001.

We then searched for the candidate target genes by computational prediction. In this way, we identified Yes-associated protein 1 (YAP) as a potential target gene for miR-181-5p (Fig. 4A). It has been reported that YAP is an oncogene having important roles in multiple tumor developments^21,22^. Increased expression of YAP can significantly facilitate the malignant transformation of cells. We next confirmed that overexpressed the level of miR-181b-5p indeed directly repressed its target gene YAP expression in osteosarcoma cells (Fig. 4B). In addition, we found that overexpressed ALKBH5 obviously silenced both mRNA and protein levels of YAP in U2OS cells (Fig. 4C), whereas suppressed ALKBH5 present elevating expression of YAP (Fig. 4D). Next, we further confirmed the effects of YAP on osteosarcoma cell growth. The silence of YAP by siRNA dramatically suppressed the proliferation, invasion, migration, and colony-forming abilities of U2OS cells (Fig. 4E–H). On the contrary, overexpression of YAP produced the opposite effects in osteosarcoma cells (Supplementary Fig. S3).

**Fig. 5:**
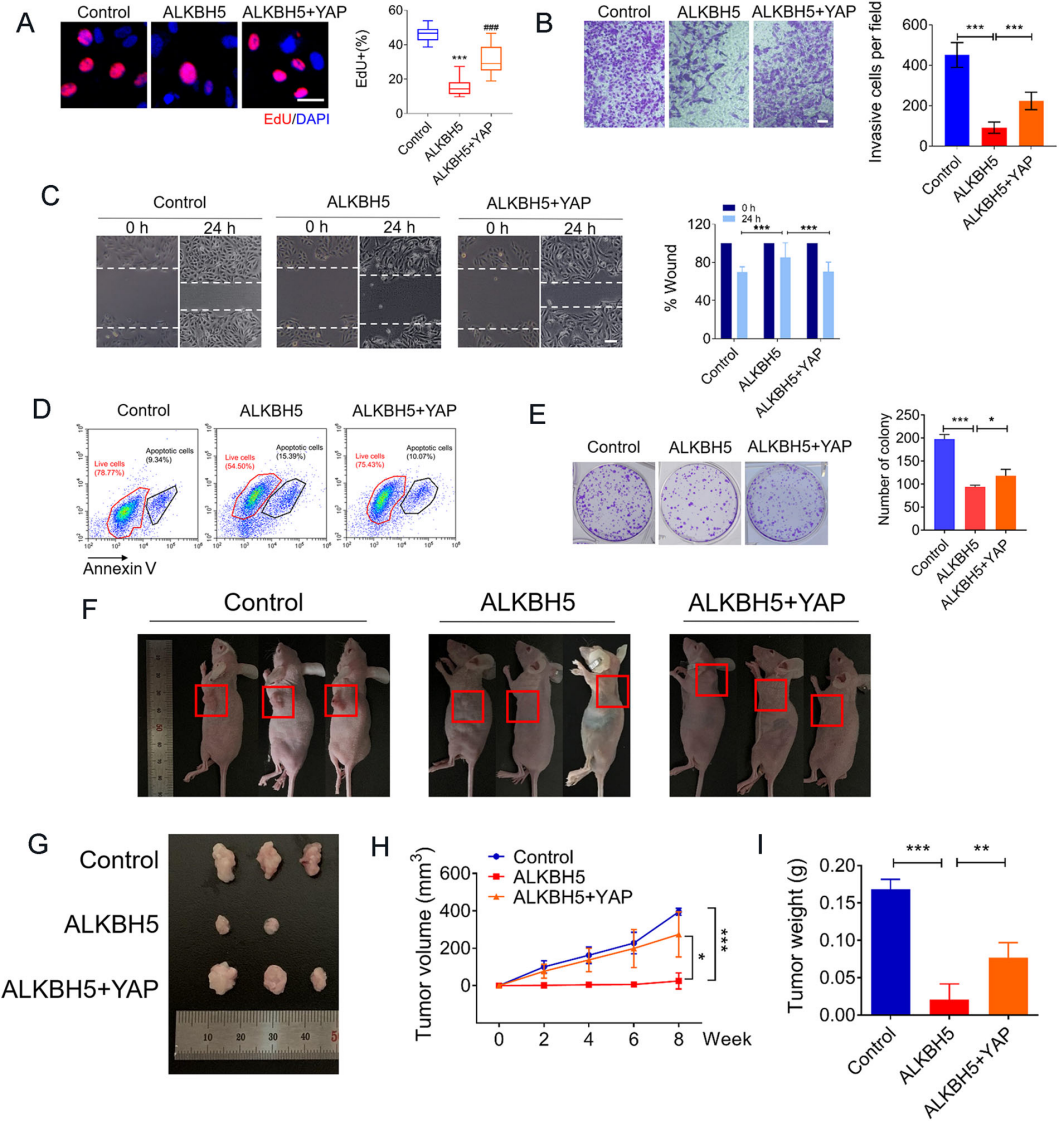
YAP abrogates the inhibition effects of ALKBH5 both on human osteosarcoma cell viability and tumor growth in the xenograft mouse model. **A** EdU staining showing the reversing effects of YAP on proliferation inhibition of U2OS cells induced by ALKBH5 overexpression (Bar: 25 μm, *n* = 5). **B** The effects of YAP on the weakening of ALKBH5 anti-invasion ability detected by Transwell assay (Bar: 150 μm, *n* = 6). **C** Cell metastasis analyzed by migration assays (Bar: 200 μm, *n* = 7). **D** Annexin V-FITC/PI staining analysis of apoptotic cells (*n* = 3). **E** Representative images of colony-formation cells (*n* = 3). **F** 143B cells transfected with empty plasmid (control), alone ALKBH5 plasmid or together with YAP plasmid, and then injected cells into female nude mice. Representative photographs of the gross 143B tumors 8 weeks after injection. The red square marks the location of the tumor (*n* = 3). **G** The image showing the comparison of the excised tumor size of 143B xenografts in nude mice. **H** Curve diagram showing the volume of the tumor measured once 2 weeks throughout the experiment. **I** Weight of tumor tissues removed from nude mice. Data are expressed as mean ± SEM. *P < 0.05; **P < 0.01; ***P < 0.001 (vs. the first group). ^###^*P* < 0.001 (vs. the second group).

To establish the relationship between ALKBH5 and YAP, we further analyzed the effects of cell growth upon co-transfection by two overexpressed plasmids of ALKBH5 and YAP in osteosarcoma cells. As shown in Fig. 5A, cell proliferation was significantly increased in the group of co-transfection by two plasmids as compared with alone ALKBH5 overexpression. Furthermore, YAP counteracted the inhibitory effects of ALKBH5 on the invasion and migration of U2OS cells (Fig. 5B, C). In line with the findings above, co-transfection of ALKBH5 and YAP markedly increased the percentage of live cells, decreased apoptotic cells (Fig. 5D), and recovered the ability of colony formation (Fig. 5E). Next, we assessed the in vivo effectiveness of ALKBH5-mediated m^6^A demethylation using an osteosarcoma xenograft mouse model. ALKBH5 overexpression reduced osteosarcoma tumor growth as evidenced by lower tumor volumes and weights, and this effect was abrogated by co-transfection of overexpressed YAP (Fig. 5F–I).

**Fig. 6:**
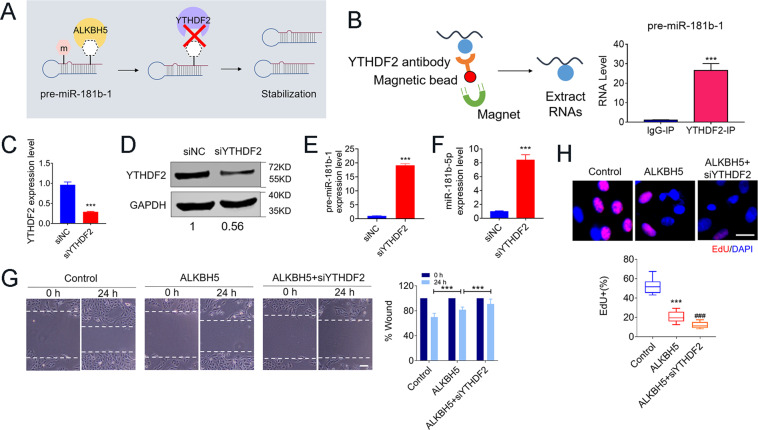
m^6^A reader YTHDF2 positively regulates pre-miR-181b-1 stabilization. **A** Schematic diagram displaying that YTHDF2 promotes pre-miR-181b-1 stabilization via competing with ALKBH5 for pre-miR-181b-1 combining. **B** RIP assay showing that the anti-YTHDF2 antibody efficiently captured miR-181-5p transcripts. **C**, **D** Transfection efficiency of YTHDF2 silencing confirmed via qRT-PCR and western blot. **E**, **F** Inhibition effects of siYTHDF2 both on pre-miR-181b-1 (**E**) and miR-181-5p expression (**F**). **G**, **H** Migration (**G**, Bar: 200 μm, *n* = 4) and proliferative abilities (**H**, Bar: 25 μm, *n* = 5) of U2OS after transfected with ALKBH5 plasmids and/or co-transfected with siYTHDF2. Data are expressed as mean ± SEM. ***P < 0.001 (vs. the first group). ^###^*P* < 0.001 (vs. the second group).

Because ALKBH5-mediated m^6^A demethylation appeared to increase pre-miR-181b-1 expression, we hypothesized that pre-miR-181b-1 is a target of YTHDF2 (Fig. 6A), the m^6^A reader protein that promotes the decay of m^6^A methylated RNAs^23^. Consistent with our hypothesis, we observed a strong enrichment of pre-miR-181b-1 in the YTHDF2-IP fractions (Fig. 6B). We then silenced YTHDF2 expression by siRNA confirmed by qRT-PCR (Fig. 6C), and western blot (Fig. 6D). SiRNA of YTHDF2 increased expression of both pre-miR-181b-1 and miR-181b-5p after transfection with siYTHDF2 in U2OS cells (Fig. 6E, F). Moreover, the tumor-suppressive effects of ALKBH5 overexpression were further enhanced by siYTHDF2 (Fig. 6G, H). The above data indicated that ALKBH5-mediated pre-miR-181b-1 m^6^A demethylation have key roles in osteosarcoma.

### Identification of YAP mRNA as a direct target of ALKBH5 in osteosarcoma

**Fig. 7:**
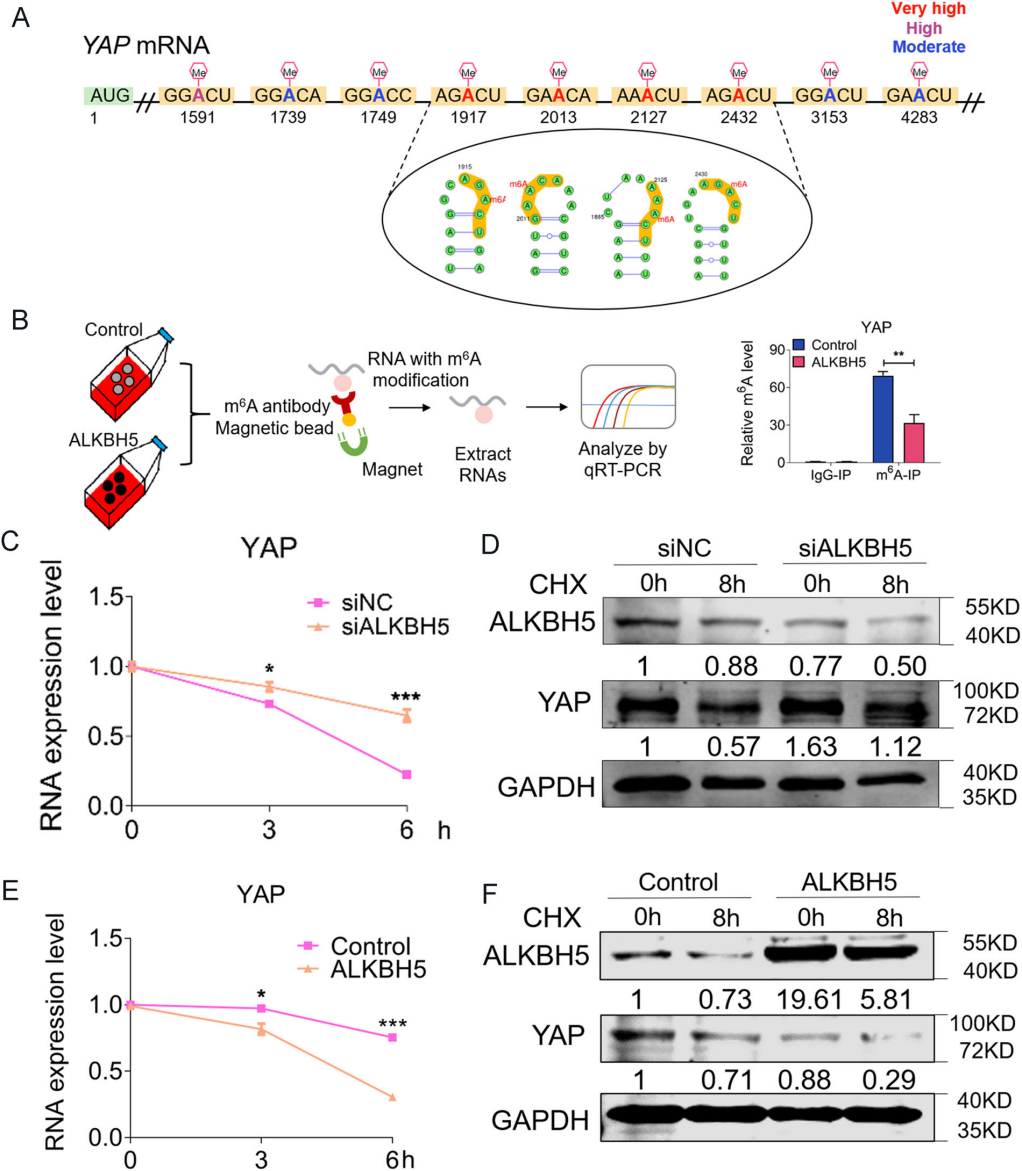
ALKBH5 directly regulates mRNA and protein stability of YAP. **A** Nine potentially m^6^A sites of YAP mRNA predicted by SRAMP program. **B** schematic diagram illustrating the procedure of gene-specific m^6^A-qPCR on left. Change of m^6^A modification in specific regions of YAP transcripts with ALKBH5 overexpression in U2OS cells on right (*n* = 4). **C** qRT-PCR showing YAP transcripts stability in ActD-treated cells after transfected with ALKBH5 siRNA. (*n* = 3). **D** YAP-protein stability after ALKBH5 silencing. Cells were harvested at 0 and 8 h after CHX treatments. **E** qRT-PCR showing YAP transcripts stability in ActD-treated cells after transfected with ALKBH5 plasmids. (*n* = 3). **F** YAP-protein stability after ALKBH5 overexpression. Cells were harvested at 0 and 8 h after CHX treatments. Data are expressed as mean ± SEM. *P < 0.05; **P < 0.01; ***P < 0.001.

Interestingly, according to a sequence-based SRAMP m^6^A modification site predictor (http://www.cuilab.cn/sramp), we observed mRNA of YAP gene carrying nine potential m^6^A modification sites (Fig. 7A and Supplementary Fig. S4). We next clarified whether ALKBH5 could directly regulate the m^6^A methylation and gene degradation of YAP. We performed gene-specific m^6^A-qPCR to test the expression of YAP. The m^6^A abundance in YAP mRNA was markedly decreased upon overexpressed ALKBH5 in the m^6^A-RIP group but was not in the IgG-RIP group (Fig. 7B). Additionally, siALKBH5 enhanced the stability of YAP mRNA in the presence of transcription inhibitor actinomycin D (ActD) as compared with siNC group in U2OS cells (Fig. 7C). Meanwhile, siALKBH5 inhibited the degradation of YAP in the presence of translation inhibitor cycloheximide (CHX) (Fig. 7D). However, as shown in Fig. 7E, F, ALKBH5 overexpression produced the opposite effects resulting in a significant decrease in the YAP-mRNA stability and an increase in the YAP-protein degradation in osteosarcoma cells.

**Fig. 8:**
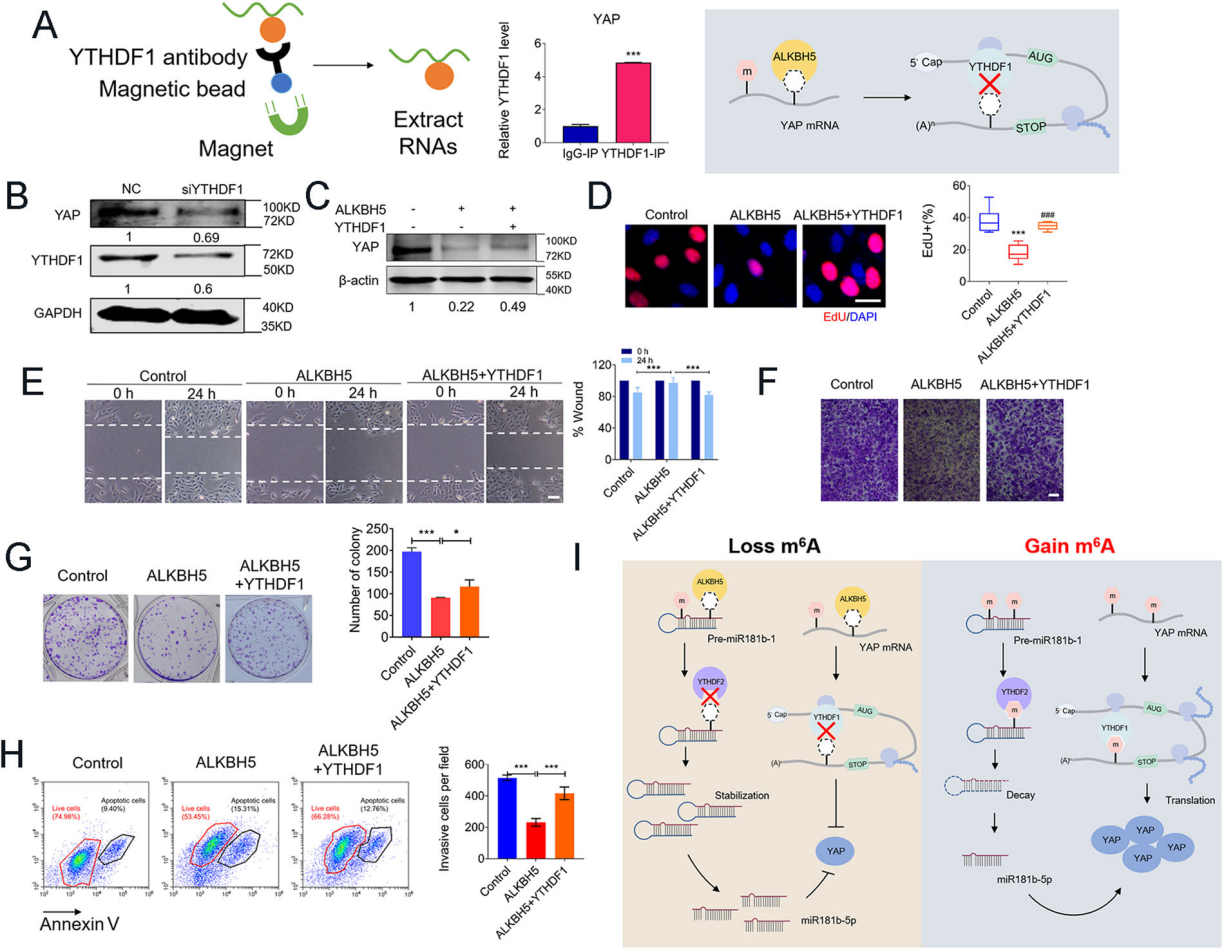
m^6^A-dependent translational enhancement of YAP is positively associated with YTHDF1. **A** RIP-qPCR analysis of the interaction between YAP with YTHDF1 in U2OS cells (left, *n* = 3). Diagram illustrating YTHDF1 replace ALKBH5 binding to YAP (right). **B** YTHDF1 knockdown impairs the translation of YAP. **C** YTHDF1 overexpression reverses the decrease of YAP caused by ALKBH5 tested via western blot. **D** EdU staining showing the reversing effects of YTHDF1 on proliferation inhibition of ALKBH5 overexpression (Bar: 25 μm, *n* = 5). **E** Cell invasion ability analyzed by migration assays at 24 h after forced expression of ALKBH5 or co-transfected with YTHDF1 plasmids. **F** The effects of YTHDF1 on the weakening of ALKBH5 anti-invasion ability detected by Transwell assay (Bar: 150 μm, *n* = 5). **G** Representative images of colony-formation cells (*n* = 3). **H** Annexin V/PI staining of U2OS cells analyzed by FACS. **I** Graphic abstract of ALKBH5 regulates the progression of osteosarcoma by mediating m^6^A modification of pre-miR-181b-1 and YAP. Data are expressed as mean ± SEM. *P < 0.05, ***P < 0.001 (vs. the first group). ^###^*P* < 0.001 (vs. the second group).

The above results revealed that ALKBH5-mediated m^6^A demethylation inhibits YAP expression, we hypothesized that methylated YAP transcripts are potential targets of YTHDF1, the m^6^A reader protein promoting the translation of methylated transcripts^24^. The abundance in YAP mRNA was markedly increased in the YTHDF1-RIP group as compared with the IgG-RIP group (Fig. 8A), suggesting that YTHDF1 can recognize the m^6^A-modification sites in YAP mRNA. Next, we found that YTHDF1 siRNA (siYTHDF1) decreased YAP-protein levels (Fig. 8B). Overexpressed YTHDF1 leads to an increased YAP in the presence of ALKBH5 overexpression in U2OS cells (Fig. 8C). As expected, upregulated the level of YTHDF1 could partially restore the inhibition effects in U2OS cell proliferation, invasion, migration, apoptosis, and colony-formation abilities (Fig. 8D–H). In line with the results for U2OS, we also observed similar effects of overexpressed YTHDF1 on cell proliferation and migration in another osteosarcoma cell line Saos2 (Supplementary Fig. S5).

## Discussion

The present study generated a number of new findings. First, m^6^A demethylase ALKBH5 expression levels are decreased, and m^6^A methylation substantially increased in human osteosarcoma. Second, ALKBH5 exerts tumor-suppressive effects as its overexpression inhibits osteosarcoma cell growth, migration, invasion, and its silence produces the opposite effects. Third, m^6^A modification of RNAs likely promotes tumor progressions induced by ALKBH5 inhibition; specifically, m^6^A methylation of pre-miR-181b-1 in the nucleus by the m^6^A mechanism causes considerable downregulation of mature miR-181b-5p in the cytoplasm, which may account at least partially for the tumor growth. Forth, our results further demonstrated that YAP is a major target gene for miR-181b-5p, and thus ALKBH5 downregulates YAP level through increasing pre-miR-181b-1/miR-181b-5p. Furthermore, we found that ALKBH5 directly inhibits m^6^A methylation of YAP, and suppresses its mRNA stability and translation thereby its cellular levels. These findings, therefore, suggest that abnormal downregulation of ALKBH5 is likely one of the mechanisms underlying the osteosarcoma (Fig. 8I). Under such a theme, we proposed that ALKBH5 is a tumor-suppressor gene, and ALKBH5 overexpression might be a new approach of replacement therapy for the treatment of human osteosarcoma.

Recent studies have demonstrated that the m^6^A demethylases were dysregulated in several malignant tumors. Li et al. provided that FTO promotes non-small cell lung cancer (NSCLC)^25^ and breast tumor progressions through increasing the expression of USP7^26^ as well as inhibiting BNIP3^27^ respectively. However, before the present study, it was unclear whether m^6^A demethylases exerted effects on osteosarcoma. We for the first time revealed that demethylase ALKBH5 overexpression could lead to downregulation of YAP level upon demethylation of its transcripts by m^6^A. In contrast, silencing of ALKBH5-induced m^6^A methylation resulting in upregulation of YAP. Consistent with our findings, Song et al. have demonstrated that m^6^A methyltransferase METTL3 was increased and promoting osteosarcoma cell progression by regulating the m^6^A level of LEF1 and activating Wnt/β-catenin signaling pathway^28^. Yet, in addition to RNA methyltransferases and demethylases, m^6^A modification exerts biological functions via interplaying with binding proteins. It has been previously reported that recognition of m^6^A mRNA sites by IGF2BP proteins enhancing mRNA stability^29^, and recognition of m^6^A by YTHDF1 result in enhanced protein synthesis^24^. Several studies have revealed the roles of m^6^A binding proteins in multiple cancer developments. For instance, SRY (sex-determining region Y)-box 2 (SOX2) was the downstream gene of METTL3, and its expression positively correlated with METTL3 and IGF2BP2 in colorectal carcinoma (CRC)^30^. Silencing YTHDF1 significantly inhibited Wnt/β-catenin pathway activity in CRC^26^. However, it also remains unclear whether these binding proteins function in osteosarcoma. Our data showed increases in both mRNA and protein expression of YAP after ALKBH5 overexpression in osteosarcoma cells. We then for the first time confirmed that YAP is a target of both YTHDF1 promoting translation of m^6^A methylated YAP transcripts.

Interestingly, we found ALKBH5 mRNA levels were much higher in Saos2 cells than in U2OS and 143B cells, while the protein levels of ALKBH5 were similar in all three cell lines. As we know, many complicated post-transcriptional mechanisms were involved in translating from mRNA into protein^31^. We assume that ALKBH5 mRNA may be modified by methylation etc. and its stability may then be different in Saos2 cells. Moreover, ALKBH5 Protein stability was also another factor. A pre-miRNA is generally exported by Exportin-5 from the nucleus to the cytoplasm where its loop structure of hairpin is further cleaved by the RNase III enzyme Dicer to generate mature a miRNA^32^. Once a mature miRNA is incorporated into the RNA-induced silencing complex (RISC), the expression of its targeted genes is repressed. Several studies have revealed the roles of miRNAs in osteosarcoma. For instance, miR-379 suppresses osteosarcoma progression by targeting PDK1^33^. MiR-491 inhibits osteosarcoma lung metastasis and chemoresistance by targeting αB-crystallin^34^. Our m^6^A-RIP-microarray data demonstrated that pre-miR-181b-1 was enriched in m^6^A-RIP fraction with ALKBH5 inhibition, which suppressed osteosarcoma tumor growth. Here, we found that pre-miRNAs can be methylated in the nucleus leading to reduced biogenesis of mature miRNAs. Yet, it remains unknown how exactly the methylation of pre-miR-181b-1 in the nucleus affects its maturation in the cytosol. This issue merits future studies in detail. A previous study has reported that recognition of m^6^A mRNA sites by YTHDF2 result in mRNA degradation^23^ mainly by starting with the shortening of the poly(A) tail and subsequent mRNA degradation. But the decay of the m^6^A-containing RNA may start by 5′-decapping or endo-cleavage^35^. However, we must admit that the deeply underlying mechanisms of YTHDF2 on pre-miRNAs are at present unknown.

It is known that YAP is a potent oncogene, which is one of the main effectors of the Hippo-YAP/TAZ tumor-suppressor pathway controlling cell proliferation and apoptosis^36^, Recent studies indicated that YAP/TAZ is essential for cancer initiation or growth of most solid tumors^37,38^. Additionally, YAP/TAZ have also been act as therapeutic targets in various cancers^38,39^. Several studies have demonstrated that YAP oncogenic function was modulated by multiple cellular factors in cancers. For instance, TNF receptor-associated factor 6 (TRAF6) promoted the migration and colony formation of pancreatic cancer cells through the regulation of YAP^40^. Neurotrophic Receptor Tyrosine Kinase 1 (NTRK1) inhibition suppressed YAP-driven transcription, cancer cell proliferation, and migration^41^. Downregulating MK5 expression inhibited the survival of YAP-activated cancer cell lines and mouse xenograft models^42^. More importantly, a study has demonstrated that YAP found to be highly expressed in both human and mouse osteosarcoma tissues^43^. Moreover, the Hippo signaling pathway has an essential role in chemoresistance as well. Of the Hippo pathways members, activation of YAP/TAZ displays resistance to chemotherapeutic drugs in tumor cells^37^. Notably, YAP has acted as a potential target for reducing osteosarcoma chemoresistance^44^. An intriguing new finding here is that YAP can be m^6^A methylated directly in the nucleus leading to enhance its mRNA stability, translation, and its cellular level in human osteosarcoma.

Collectively, our results for the first time suggest that ALKBH5 is an anti-tumor factor or a pro-apoptotic factor, acting at least partially by suppressing YAP expression through dual mechanisms with direct m^6^A methylation of YAP and indirect downregulation of YAP level due to methylation of pre-miR-181b-1. We have also demonstrated that pre-miRNAs could be methylated by the ALKBH5 mechanism in the nucleus leading to significant alterations of their maturation in the cytosol.

## Material and methods

### Cell culture and treatment

Human osteosarcoma cell line U2OS was cultured in Dulbecco’s modified Eagle medium (DMEM) (Life Technologies Corporation, California, USA) supplemented with 10% fetal bovine serum (FBS) (Biological Industries, Israel). Saos2 was grown in McCOY’S 5A medium (HyClone, California, USA) supplemented with 15% fetal bovine serum. 143B was cultivated in RPMI Medium 1640 basic medium (ThermoFisher Scientific, Massachusetts, USA). While human osteoblasts (hOB) cell line hFOB1.19 was cultured in DMEM/F-12 (1:1) basic medium (ThermoFisher Scientific, Massachusetts, USA) supplemented with 10% fetal bovine serum. All cell lines were maintained in an incubator at 37 °C in an atmosphere containing 5% CO_2_, except hFOB1.19 cells, which were incubated at 33.5 °C. All cell lines were tested for mycoplasma contamination. Cycloheximide (CHX, Cat# HY-12320, MedChemExpress, China) was treated cells for 0 and 8 h by adding into the medium at 100 μg/mL before harvesting.

### M^6^A enzyme-linked immunosorbent assay (ELISA)

The m^6^A RNA methylation assay kit (Cat#ab185912; Abcam, Cambridge, UK) was used to measure the m^6^A content in total RNAs following the manufacture’s protocol. Briefly, 400 ng RNAs were coated on assay wells, and then incubated at 37 °C for 90 min. 50 μL capture antibody solution and 50 μL detection antibody solution was then added to assay wells separately incubated for 60 and 30 min at room temperature (RT). The m^6^A levels were quantified using a microplate reader at a wavelength of 450 nm.

### Immunofluorescence (IF)

2 × 10^5^ cells were cultured in a glass-bottom cell culture dish for 24 h, and washed with PBS three times. Fixed the cells with 4% paraformaldehyde at RT for 15 min and then washed the cells three times with PBS. Permeabilized cells with 0.3% Triton X-100 (Sigma-Aldrich) for 15 min, and then blocked with goat serum. After washing twice with PBS, treated the cells with primary antibodies of m^6^A (1:200 dilution, Synaptic Systems, Germany) and ALKBH5 (1:200 dilution, Millipore, Billerica, USA) and incubated at 4 °C overnight. Finally, incubated cells with secondary antibody at RT. Fluorescent images were visualized using a confocal microscope (Fv10i).

### RNA extraction

Total RNA was isolated using the miRNeasy Mini Kit (Cat# 217004; QIAGEN, Germany) according to the manufacturer’s protocol. Briefly, cells were disrupted and homogenized with QIAzol Lysis Reagent at RT for 5 min. Then chloroform was added to the samples followed by vigorous shaking for 15 s. After centrifugation at 12,000 × *g* at 4 °C for 15 min, the upper aqueous phase was transferred to a new collection tube and mixed with ethanol thoroughly. The sample of 700 μL was pipetted into an RNeasy Mini column followed by centrifugation at 8000 × *g* at RT for 15 s to discard the flow-through. After washing sequentially with buffer RWT and buffer RPE, RNA was dissolved with RNase-free water.

### Quantitative real-time PCR

Total RNA sample of 500 ng was reverse transcribed to cDNA using High Capacity cDNA Reverse Transcription Kit (Cat# 00676299; ThermoFisher Scientific, Waltham, USA). Amplification and detection were performed using 7500HT Fast Real-Time PCR System (Applied Biosystems) with SYBR Green PCR Master Mix (Cat# 31598800; Roche). GAPDH was used as an endogenous control. For miRNA analyses, U6 was used as an internal standard control. miRNA primers were obtained from RiboBio (Guangzhou, China). Reactions were run in triplicate. The primer pairs used in our PCR analysis are: Forward/Reverse primer sequence (5′–3′)

$${\mathrm{METTL}}3 - {\mathrm{F}}\!:\!{\mathrm{CGACGGAAGTATCGCTTGTCA}}$$

$${\mathrm{METTL}}3 - {\mathrm{R}}\!:\!{\mathrm{TTCACCGAGGTCAGCAGTATG}}$$

$${\mathrm{METTL}}14 - {\mathrm{F}}\!:\!{\mathrm{GTCTTAGTCTTCCCAGGATTGTTT}}$$

$${\mathrm{METTL}}14 - {\mathrm{R}}\!:\!{\mathrm{AATTGATGAGATTGCAGCACC}}$$

$${\mathrm{FTO}} - {\mathrm{F}}\!:\!{\mathrm{GACCTGTCCACCAGATTTTCA}}$$

$${\mathrm{FTO}} - {\mathrm{R}}\!:\!{\mathrm{AGCAGAGCAGCATACAACGTA}}$$

$${\mathrm{ALKBH}}5 - {\mathrm{F}}\!:\!{\mathrm{ACTGAGCACAGTCACGCTTCC}}$$

$${\mathrm{ALKBH}}5 - {\mathrm{R}}\!:\!{\mathrm{GCCGTCATCAACGACTACCAG}}$$

$${\mathrm{WTAP}} - {\mathrm{F}}\!:\!{\mathrm{TTACCTTTCCCACTCACTGCT}}$$

$${\mathrm{WTAP}} - {\mathrm{R}}\!:\!{\mathrm{AGATGACTTTCCTTCTTCTCCA}}$$

$${\mathrm{YAP}} - {\mathrm{F}}\!:\!{\mathrm{TGCGTAGCCAGTTACCA}}$$

$${\mathrm{YAP}} - {\mathrm{R}}\!:\!{\mathrm{GGTGCCACTGTTAAGGA}}$$

$${\mathrm{YTHDF}}1 - {\mathrm{F}}\!:\!{\mathrm{ACCTGTCCAGCTATTACCCG}}$$

$${\mathrm{YTHDF}}1 - {\mathrm{R}}\!:\!{\mathrm{TGGTGAGGTATGGAATCGGAG}}$$

$${\mathrm{YTHDF}}2 - {\mathrm{F}}\!:\!{\mathrm{CAGGCATCAGTAGGGCAACA}}$$

$${\mathrm{YTHDF}}2 - {\mathrm{R}}\!:\!{\mathrm{TTATGACCGAACCCACTGCC}}$$

$${\mathrm{GAPDH}} - {\mathrm{F}}\!:\!{\mathrm{AGCCACATCGCTCAGACAC}}$$

$${\mathrm{GAPDH}} - {\mathrm{R}}\!:\!{\mathrm{GCCCAATACGACCAAATCC}}$$

### Tissue microarrays (TMAs) and Immunohistochemistry (IHC) analysis

Osteosarcoma tissue microarrays were purchased from the Bioaitech Company (Xi’an, China), comprised of 2 normal bone tissues, 100 malignant osteosarcoma cores. The slide was baked at 60 °C for 30 min and then followed by antigen retrieval with tris-EDTA buffer (pH 9.0), medium heat for 10 min to boil, cease-fire for 5 min, and washed with PBS for 5 min ×3 times. Endogenous peroxidase was blocked with 3% H_2_O_2_-methanol at RT for 10 min and washed with PBS for 5 min × 3 times. The sections were added normal non-immune animal serum at RT for 10 min and then removed the serum and added a drop of anti-ALKBH5 (1:300) at 4 °C overnight. Then it was washed with 0.1% tween-20 PBS for 5 min × 3 times. Biotin-labeled sheep anti-mouse/rabbit IgG was added and incubated at RT for 10 min followed by washing with 0.1% tween-20 PBS for 5 min × 3 times. Streptomyces anti-biotin protein-peroxidase was added and incubated at RT for 10 min. DAB working solution was incubated for 5 min and stopped by distilled water washing. After hematoxylin re-staining, washing and differentiation, the slide returned to be blue with full washing followed with regular dehydration transparent and being sealed by neutral gum. The percentage of ALKBH5 positive cells were counted in 5 (×400) high-power fields (upper, lower, left, right, and middle) under the microscope, and the mean values were then calculated.

### Western blot analysis

The western blot analysis was described as previously^45^. Briefly, osteosarcoma cell lines were lysed in cell lysis buffer (Cat# P0013B; Beyotime Biotechnology, Shanghai, China) supplemented with PMSF protease inhibitor on ice for 30 min, followed by centrifuging at 13,500 × *g* at 4 °C for 15 min. The protein concentration was quantified using a BCA Protein Assay Kit (Cat# P0010S; Beyotime Biotechnology) following the manufacturer’s instructions. The protein sample (50 µg) was separated on a polyacrylamide gel and transferred to a nitrocellulose membrane and then blocked with 5% fat-free dry milk at RT for 1 h. Then, the membrane was incubated with a rabbit anti-YAP antibody (1:1000; Cat# D8H1X; Cell Signaling Technology), a rabbit anti-ALKBH5 antibody (1:1000; Cat# ABE547; Millipore, Billerica, USA), a rabbit anti-YTHDF1 antibody (1:1000; Cat# 17479-1-AP; Proteintech, Wuhan, China), a rabbit anti-YTHDF2 antibody (1:1000; Cat# 17479-1-AP; Proteintech), a mouse anti-Tubulin antibody (1:1000; Cat# abs830032; Absin, Shanghai, China), a mouse anti-β-actin antibody (1:1000; Cat# sc-47778; Santa Cruz Biotechnology, Dallas, Texas, United States), a mouse anti-GAPDH antibody (1:500; Cat# abs830030; Absin) at 4 °C overnight. A secondary incubation step was carried out with monoclonal anti-rabbit IgG (1:5000; Cat# ab97051; Abcam) or monoclonal anti-mouse IgG (1:5000; Cat# ab6789; Abcam) at RT for 1 h. Western blot bands were imaged by odyssey CLx and quantified with LI-COR Image Studio Software (LI-COR Biosciences, Lincoln, NE, USA).

### siRNAs transfection

Cells were transfected to knockdown the expression of ALKBH5, YAP, YTHDF1, YTHDF2, and miR-181b-5p using Lipofectamine TM 3000 Transfection Reagent (Cat# L3000-015; Invitrogen, California, USA) according to the manufacturer’s instructions. Briefly, 2 × 10^5^ cells were seed in a 6-well plate and they will be 70% confluent at the time of transfection. 6 μL Lipofectamine TM 3000 reagent and 40 nM siRNA (Gene Pharma, Shanghai, China) were diluted in 125 μL Opti-MEM (Cat# 31985-070; gibco, Grand Island, USA) medium respectively. After Mix and incubation for 2 min separately, these two regents were then mixed and incubated for another 10 min and then added it to cells. Subsequent experimental measurements were performed 24 h after transfection. The siRNAs and miR-181b-5p inhibitor sequence used are as following:

$${\mathrm{ALKBH}}5\;{\mathrm{siRNA}}\!:\!5^\prime - {\mathrm{CUGCGCAACAAGUACUUCUTT}} - 3^\prime$$

$${\mathrm{YAP}}\;{\mathrm{siRNA}}\!:\!5^\prime - {\mathrm{GGUGAUACUAUCAACCAAATT}} - 3^\prime$$

$${\mathrm{YTHDF}}1\;{\mathrm{siRNA}}\!:\!5^\prime - {\mathrm{CCUGCUCUUCAGCGUCAAUTT}} - 3^\prime$$

$${\mathrm{YTHDF}}2\;{\mathrm{siRNA}}\!:\!5^\prime - {\mathrm{AAGGACGUUCCCAAUAGCCAATT}} - 3^\prime$$

$${\mathrm{miR}} - 181{\mathrm{b}} - 5{\mathrm{p}}\;{\mathrm{inhibitor}}\;\left( {{\mathrm{AMO}}} \right)\!:\!5^\prime - {\mathrm{ACCCACCGACAGCAAUGAAUGUU}} - 3^\prime$$

### Plasmid transfection

ALKBH5- and YAP-carrying plasmid for overexpression were constructed by Cyagen (Suzhou, China). The YTHDF1-carrying plasmid was obtained from Genechem (Shanghai, China). miR-181b-5p mimic was obtained from Gene Pharma. Cells were transfected with 500 ng plasmid using Lipofectamine TM 3000 Transfection Reagent according to the manufacturer’s protocols. Cells were collected 24 h after transfection. The miR-181b-5p mimic sequence used is 5′-AACAUUCAUUGCUGUCGGUGGGU-3′.

### Ethynyl-2-deoxyuridine (EdU) staining assay

EdU Apollo DNA in vitro kit (Ribobio, Guangzhou, China) was used to detect cell proliferation. Cells were plated into a glass-bottom cell culture dish (NEST, Hong Kong, China) at a density of 2.0 × 10^5^. Briefly, cells were fixed with 4% paraformaldehyde (m/v) for 30 min, and followed by incubation of 30 μM EdU at 37 °C for 90 min. After permeabilized in 0.5% Triton X-100, the Apollo staining solution was added into the cell culture medium for 30 min in the dark. Finally, the cells were incubated with 20 μg/mL 4′,6-diamidino-2-phenylindole (DAPI) for 10 min. The EdU index (%) was the average ratio of the number of EdU-positive cells over total cells in five randomly selected areas under the confocal laser scanning microscope (FV10i).

### Invasion assays

A 24 mm Transwell® chambers (Corning #3412, USA) was used to detect cell invasive abilities according to the manufacturer’s protocol. 5 × 10^4^ cells infected with plasmid or siRNAs were resuspended in 200 μL serum-free DMEM medium, and seeded in the upper chamber. DMEM medium contained with 10% FBS was added into the lower chamber. After 24 h, cells migrated through the membrane were stained with 0.1% crystal violet (Beyotime Biotechnology, China) for 15 min and counted using light microscopy (ECLIPSE TS100, Nikon).

### Migration assays

Cells were plated into 6-well culture plates at a density of 2.5 × 10^5^ cells/mL. When the confluence of cells reached 70%, a wound was created by scraping the cells with a 200 μL pipette tip. Cells were washed with PBS and then transfected with siRNAs or plasmid. Images were captured at 0 and 24 h after wounding with standard light microscopy (ECLIPSE TS100, Nikon, Japan). The wound area was measured using ImageJ software (National Institutes of Health (NIH), USA).

### Colony-formation assay

Cells transfected with targeted siRNA or plasmid were seeded in a six-well plate with a concentration of 1000 cells per well and cultured in a humidified atmosphere containing 5% CO_2_ at a constant temperature of 37 °C to form colonies. Two weeks later, cells were fixed and stained with 100% methanol and 0.1% crystal violet for 20 min, separately. Colonies were air-dried and counted. The experiments were repeated three times.

### Flow cytometry

FITC Annexin V Apoptosis Detection Kit I (BD Pharmingen, Cat# 556547) was used to detected apoptosis according to the manufacturer’s instructions. Briefly, cells were moistened and washed with precooled PBS twice, and then centrifugated at 1500 rpm for 5 min. Cells were diluted with 300 μL 1× Binding Buffer, 5 μL FITC-labeled Annexin V and 5 μL propidium iodide (PI) were added in cell suspension and stained for 15 min. Data were analyzed with CytExpert software.

### Human m^6^A epitranscriptomic microarray analysis

Total RNA samples were extracted from ALKBH5-overexpressed U2OS cells and the corresponding negative control cells. The samples were incubated with m^6^A antibody for immunoprecipitation (IP). The modified RNAs were eluted from the immunoprecipitated magnetic beads as the “IP”, and the unmodified RNAs were recovered from the supernatant as “Sup”. The RNAs were labeled with Cy5 and Cy3, respectively, and designated as cRNAs in separate reactions using Arraystar Super RNA Labeling Kit (Arraystar, Rockville, USA). The cRNAs were combined and hybridized onto Arraystar Mouse Epitranscriptomic Microarray (8×60K, Arraystar). After washing the slides, the arrays were scanned in two-color channels by an Agilent Scanner G2505C. Raw intensities of IP (Cy5-labeled) and Sup (supernatant, Cy3-labeled) were normalized with an average of log_2_-scaled Spike-in RNA intensities. After Spike-in normalization, the probe signals having Present (P) or Marginal (M) QC flags in at least 1 out of 2 samples were retained as “All Targets Value” in the excel sheet for determination of m^6^A methylation level and m^6^A quantity. m^6^A methylation level was calculated as the percentage of modification based on the IP (Cy5-labeled) and Sup (Cy3-labeled) normalized intensities. m^6^A quantity was calculated to indicate the degree of m^6^A methylation of RNAs based on the IP-normalized intensities. Differentially m^6^A-methylated RNAs were identified by filtering with the fold changes of >1.2.

### m^6^A quantification

Quantification of m^6^A RNA methylation was detected by m^6^A RNA methylation assay kit (Cat# ab185912; Abcam, Cambridge, UK) following the manufacture’s protocol. Total RNA samples of 400 ng for each group were used to determine the percentage of m^6^A. The absorbance was measured at 450 nm using a microplate reader and the percentage of m^6^A in total RNA was calculated using the following equation:

$${\mathrm{m}}^6{\mathrm{A}}\% = \frac{{\left( {{\mathrm{SampleOD}} - {\mathrm{NCOD}}} \right) \div S}}{{\left( {{\mathrm{PCOD}} - {\mathrm{NCOD}}} \right) \div P}} \times 100\%$$

where *S* represents the amount of input RNA sample in ng, and *P* the amount of input of positive control in ng.

### Methylated RNA immunoprecipitation (MeRIP)-qPCR

The Magna MeRIP Kit (Cat# CR203146; Millipore, Massachusetts, USA) was used according to the manufacturer’s instructions to examine m^6^A modification on genes. Cells were harvested prior to washing with ice-cold PBS twice and subsequently collected by centrifugation at 1500 rpm at 4 °C for 5 min. Having removed the supernatant, the cells were mixed with 100 μL RIP lysis buffer and incubated with the lysate on ice for 5 min. The cell preparation was then stored at −80 °C. m^6^A antibody (5 μg) was added to a tube containing magnetic beads, followed by rotation at RT for 30 min. The beads were washed with RIP wash buffer twice and resuspended in 900 μL RIP immunoprecipitation buffer mixed with 100 μL cell lysate followed by centrifugation at 14,000 rpm at 4 °C for 10 min. After rotation at 4 °C overnight, the beads were washed with high salt buffer, followed by extraction with RIP wash buffer. RNA enrichment was analyzed by qRT-PCR.

### RNA stability assay

Cells were seed in a 6-well plate and transfected with desired constructs as described above. After 24 h transfection, cells were treated with actinomycin D (5 μg/mL; Cat# HY-17559; SIGMA-ALDRICH) for 0, 3, and 6 h before collection. Total RNAs were isolated for qRT-PCR analysis.

### Xenograft tumorigenesis model

Three-week-old BABL/c female nude mice were purchased from Beijing Vital River Laboratory Animal Technology Limited Company (Beijing, China) and randomized into three groups. 5 × 10^6^ 143B cells were subcutaneously injected in mice, and the tumor volume was assessed every 2 weeks. Eight weeks after injection, the animals were killed. The xenograft tumors were harvested and the tumor volumes were calculated by the standard formula: length × width^2^/2. All animal studies were approved by the Animal Care and Use Committee of Harbin Medical University.

### Statistical analysis

The investigators were blinded to the group allocation during the experiments of the study. Data are expressed as mean ± SEM. Statistical analysis was performed using GraphPad Prism5 software and analyzed with Student’s *t*-test (two-tailed). All experiments were independently repeated at least three times, with similar results obtained. *P < 0.05; **P < 0.01; ***P < 0.001.

## Supplementary information

Figure S1

Figure S2

Figure S3

Figure S4

Figure S5

Supplementary Figure Legends
