# Supplementary figures and images for: ALKBH5 suppresses tumor progression via an m6A-dependent epigenetic silencing of pre-miR-181b-1/YAP signaling axis in osteosarcoma
Source: Cell Death Dis. 2021 Jan 11;12(1):60. doi: 10.1038/s41419-020-03315-x (PMC7801648; doi:10.1038/s41419-020-03315-x)

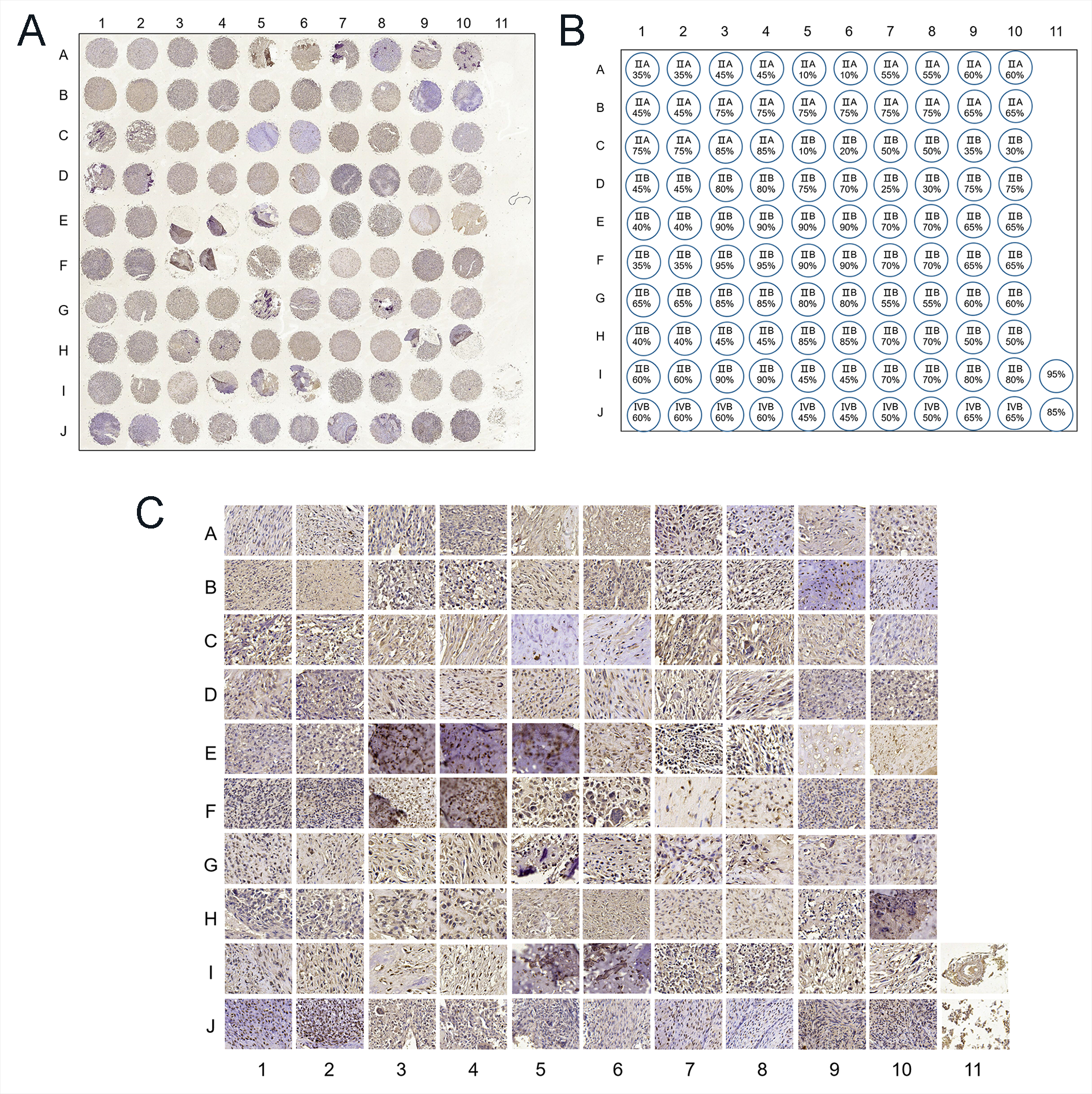

Supplement: Supplementary file 1 — Figure S1 [file 41419_2020_3315_MOESM1_ESM.tif]

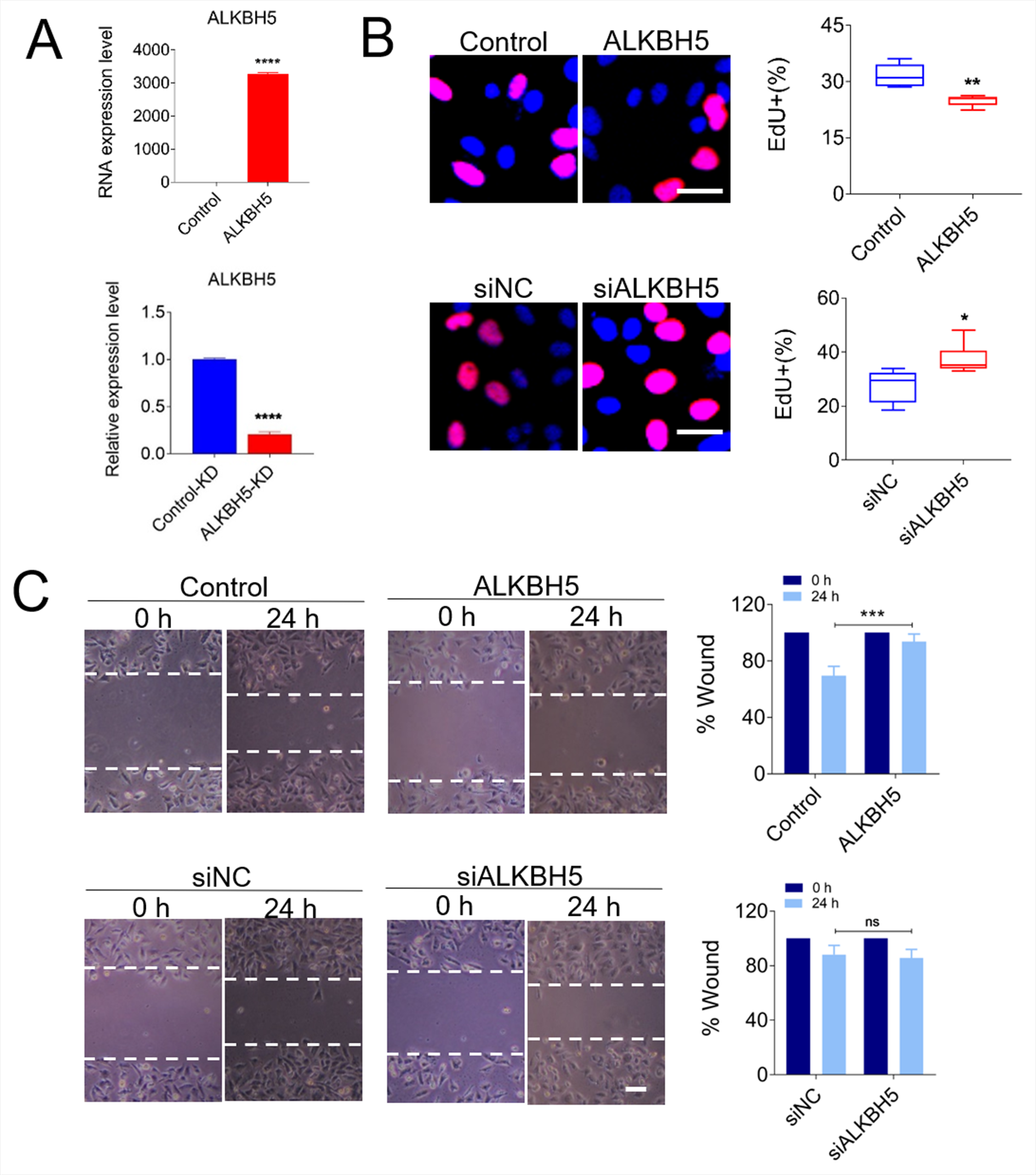

Supplement: Supplementary file 2 — Figure S2 [file 41419_2020_3315_MOESM2_ESM.tif]

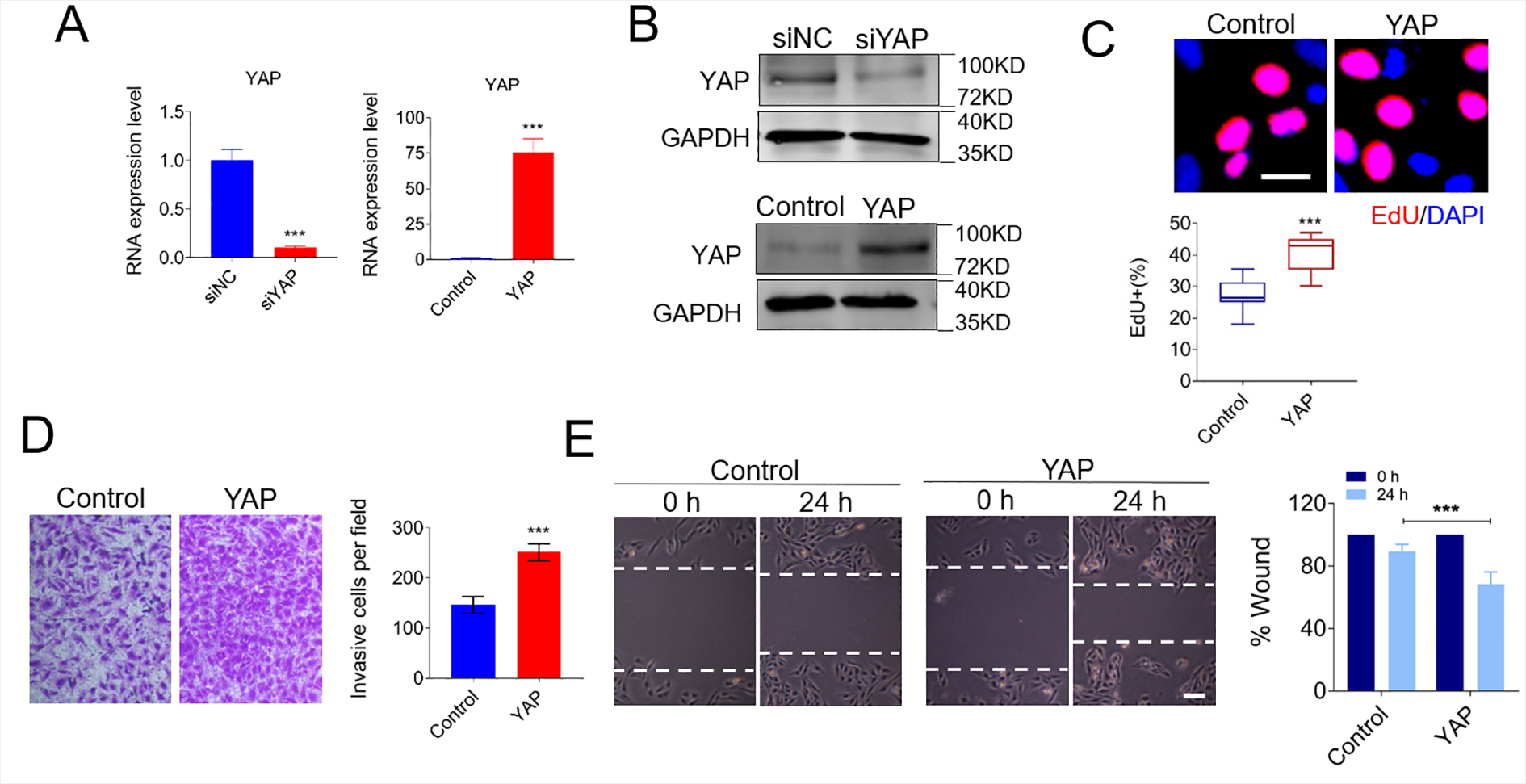

Supplement: Supplementary file 3 — Figure S3 [file 41419_2020_3315_MOESM3_ESM.tif]

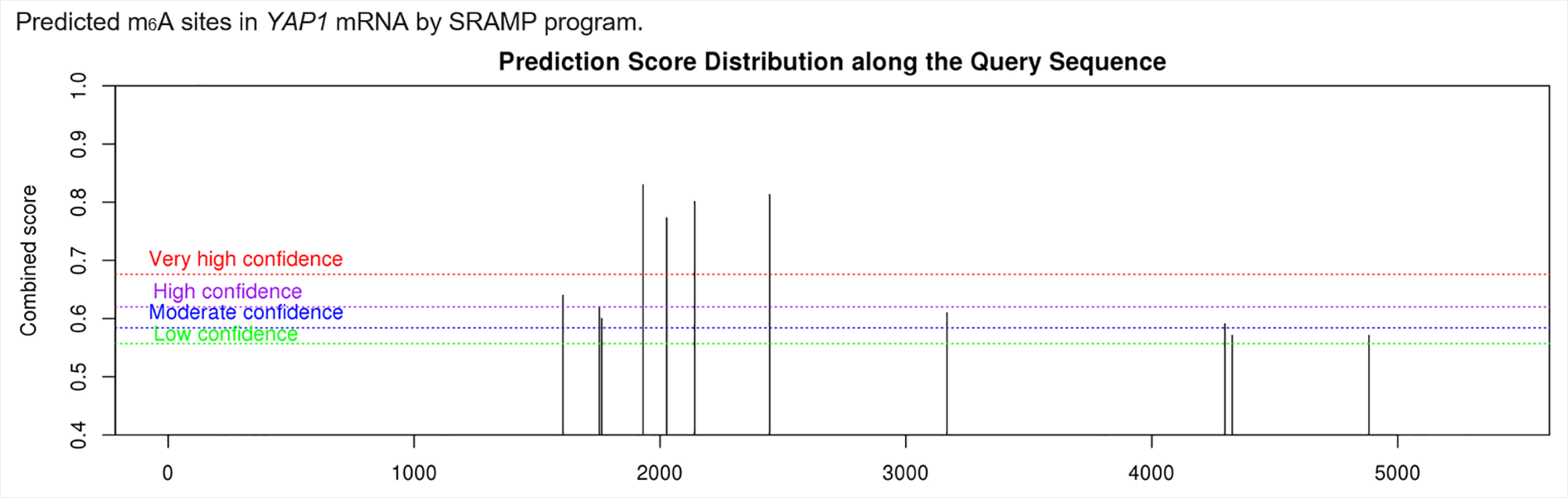

Supplement: Supplementary file 4 — Figure S4 [file 41419_2020_3315_MOESM4_ESM.tif]

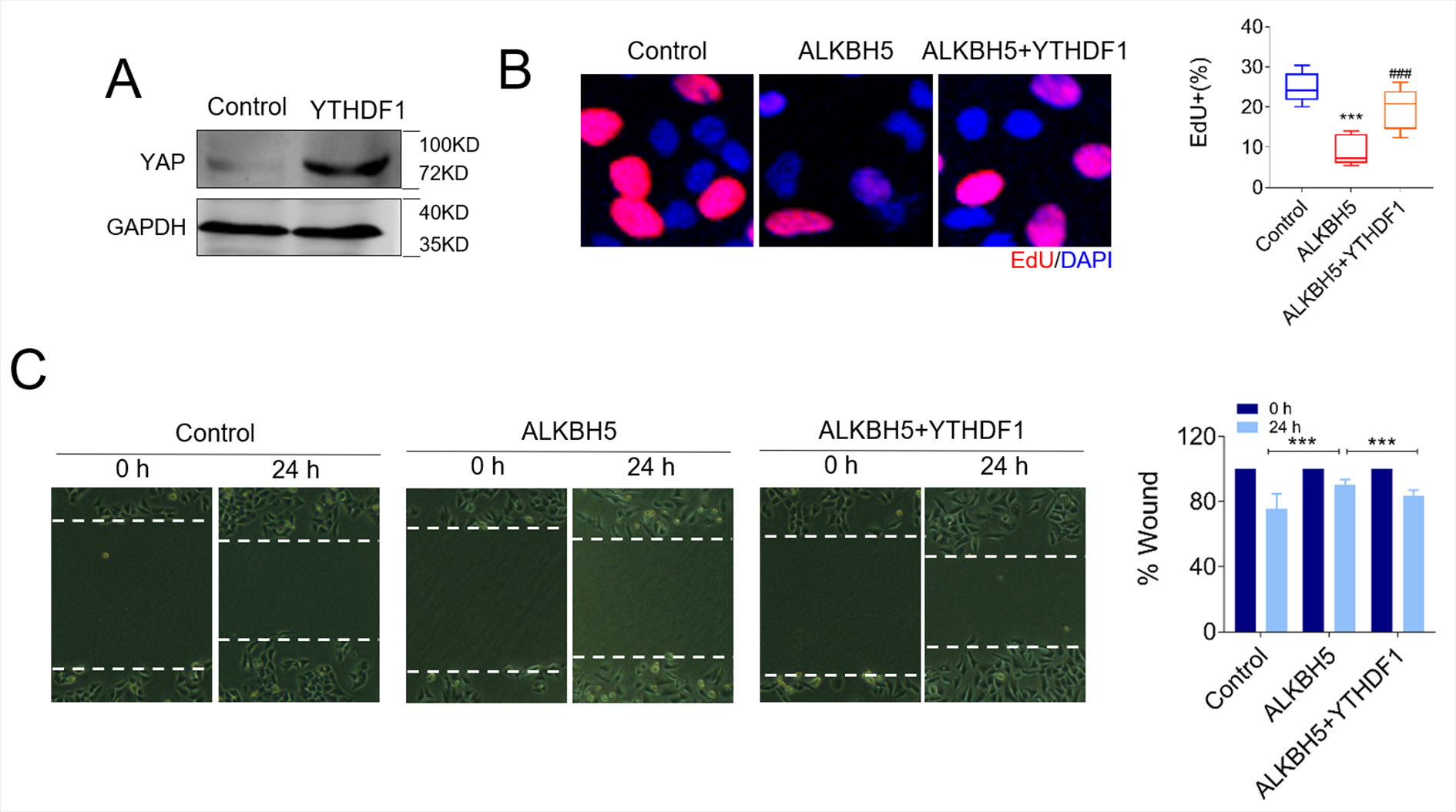

Supplement: Supplementary file 5 — Figure S5 [file 41419_2020_3315_MOESM5_ESM.tif]
