# Supplementary material for: ALKBH5 suppresses tumor progression via an m6A-dependent epigenetic silencing of pre-miR-181b-1/YAP signaling axis in osteosarcoma
Source: Cell Death Dis. 2021 Jan 11;12(1):60. doi: 10.1038/s41419-020-03315-x (PMC7801648; doi:10.1038/s41419-020-03315-x)
Supplement: Supplementary file 6 — Supplementary Figure Legends [file 41419_2020_3315_MOESM6_ESM.docx]

**Figure S1. Immunohistochemical expression of ALKBH5 in osteosarcoma.** (A) Representative tissue microarray containing 100 malignant osteosarcoma cores and 2 normal tissues cores. (B) The table below shows the stage of osteosarcoma and the percentage of ALKBH5 positive cells number at the corresponding location. (C) Representative images of the corresponding location cores in tissue microarray.

**Figure S2. The role of ALKBH5 on Saos2 cell proliferation and cell apoptosis.** (A) Transfection efficiency of ALKBH5 plasmid (left) or siRNA (right) verified by qRT-PCR. (n=3) (B) Change of Saos2 cell proliferation ability tested by EdU staining (Bar: 25 μm, n=5) after transfected with ALKBH5 plasmid or siRNA. (C) Effects of ALKBH5 overexpression or knockdown on Saos2 cell migration. (n=4) Data are expressed as mean ± SEM. *P < 0.05; **P < 0.01; ***P < 0.001; ns, no significance.

**Figure S3. Biology impact of forced expression of YAP in U2OS cells.** (A and B) Efficiency of YAP knockdown or forced expression verified by qRT-PCR (A, n=3) and Western-blot (B) in U2OS cells. (C) Inhibitory effects of YAP forced expression on U2OS cell proliferation evaluated by EdU staining (Bar: 25 μm, n=5). (D) Trans-well assays for the invasion ability of U2OS cells. (Bar: 150 μm, n=4) (E) Wound-healing assay showing the migration ability of U2OS cells. (Bar: 200 μm, n=4) Data are expressed as mean ± SEM. ***P < 0.001.

**Figure S4. The potential m^6^A modification sites of YAP1 mRNA by SRAMP program.**

**Figure S5. YTHDF1 regulates the m^6^A-dependent translational enhancement of YAP in Saos2 cells line.** (A) Effects of YTHDF1 overexpression on the translation of YAP in Saos2 cells. (B) EdU staining detecting the impact of YTHDF1 on anti-proliferation ability of ALKBH5 overexpression in Saos2 cells. (Bar: 25 μm, n=9) (C) YTHDF1 dismiss the inhibitory action of ALKBH5 on cell migration (Bar: 200 μm, n=5) of Saos2 cells. Data are expressed as mean ± SEM. ***P < 0.001; ^###^P < 0.001.
